# Toxicity of *Rhododendron anthopogonoides* Essential Oil and Its Constituent Compounds towards *Sitophilus zeamais*

**DOI:** 10.3390/molecules16097320

**Published:** 2011-08-25

**Authors:** Kai Yang, Yu Xin Zhou, Cheng Fang Wang, Shu Shan Du, Zhi Wei Deng, Qi Zhi Liu, Zhi Long Liu

**Affiliations:** 1 Department of Entomology, China Agricultural University, Haidian District, Beijing 100193, China; 2 Institute of Chinese Materia Medica, Henan University, Kaifeng 475001, China; 3 State Key Laboratory of Earth Surface Processes and Resource Ecology, Beijing Normal University, Beijing 100875, China; 4 Analytical and Testing Center, Beijing Normal University, Beijing 100875, China

**Keywords:** *Rhododendron anthopogonoides*, *Sitophiluszeamais*, contact toxicity, fumigant, 4-phenyl-2-butanone, essential oil composition

## Abstract

The screening of several Chinese medicinal plants for insecticidal principles showed that essential oil of *Rhododendron anthopogonoides* flowering aerial parts possessed significant toxicity against maize weevils, *Sitophilus zeamais*. A total of 37 components were identified in the essential oil and the main constituents of the essential oil were 4-phenyl-2-butanone (27.22%), nerolidol (8.08%), 1,4-cineole (7.85%), caryophyllene (7.63%) and γ-elemene (6.10%), followed by α-farnesene (4.40%) and spathulenol (4.19%). Repeated bioactivity-directed chromatographic separation on silica gel columns led us to isolate three compounds, namely 4-phenyl-2-butanone, 1,4-cineole, and nerolidol. 4-Phenyl-2-butanone shows pronounced contact toxicity against *S. zeamais* (LD_50_ = 6.98 μg/adult) and was more toxic than either 1,4-cineole or nerolidol (LD_50_ = 50.86 μg/adult and 29.30 μg/adult, respectively) against the maize weevils, while the crude essential oil had a LD_50_ value of 11.67 μg/adult. 4-Phenyl-2-butanone and 1,4-cineole also possessed strong fumigant toxicity against the adults of *S. zeamais* (LC_50_ = 3.80 mg/L and 21.43 mg/L) while the crude essential oil had a LC_50_ value of 9.66 mg/L.

## 1. Introduction

Currently, control of stored product insects relies heavily on the use of synthetic insecticides and fumigants, which has led to problems such as disturbance of the environment, increasing application costs, pest resurgence, pest resistance to pesticides and lethal effects on non-target organisms in addition to direct toxicity to the users [1]. Thus, there is a considerable interest in developing natural products that are relatively less damaging to mammalian health and the environment than existing conventional pesticides, as alternatives to non-selective synthetic pesticides to control the pests of medical and economic importance [2,3]. In recent years, various workers have been concentrating their efforts on the search for natural products as an alternative to conventional insecticides and fumigants, as well as the re-evaluation of traditional botanical pest control agents [4,5,6,7,8,9,10,11].

Botanical pesticides have the advantage of providing novel modes of action against insects that can reduce the risk of cross-resistance, as well as offering new leads for the design of target-specific molecules [2]. During a screening program for new agrochemicals from Chinese medicinal herbs and local wild plants, the essential oil derived from flowering aerial parts of *Rhododendron anthopogonoides* Maxim. (Family: Ericaceae) was found to possess strong insecticidal activity against the maize weevil, *Sitophilus zeamais* Motsch. *R. anthopogonoides* is a shrub, growing on the damp sides of mountains and widely distributed in northwest China, especially in Sichuan, Qinghai, and Gansu provinces [12]. Its flowers, leaves, and twigs are used as traditional Chinese folk medicine for treating chronic bronchitis and coronary heart disease [13]. Investigations have shown that the crude drug contains monterpenoids, sesquiterpenoids, triterpenoids, flavonoids, steroids, coumarins, lignans, cerebrosides, tetracyclic chromane derivatives, tannins, and alkaloids [14,15,16,17,18,19,20,21]. The essential oil of this medicinal herb has also been investigated in the previous studies [22,23,24,25,26]. The essential oil of *R. anthopogonoides* aerial parts was demonstrated to inhibit growth of bacteria (*Bacillus subtilis*, *Escherichia coli*, *Proteus vulgaris*, and *Staphyloccocus aureus*) [23]. Moreover, the essential oil of *R. anthopogonoides* exhibited strong antifeedant, stomach poison, contact toxicity, and growth inhibitory effects to the larvae of grassland caterpillar (*Gynaephora menyuanenis*) [27]. However, no compounds active against stored product insects were isolated from the essential oil of *R. anthopogonoides* so far. In this paper, we report the isolation of three components derived from this essential oil active against the maize weevil and the chemical composition and insecticidal activities of the essential oil of *R. anthopogonoides* were also determined.

## 2. Results and Discussion

### 2.1. Isolated Bioactive Compounds

**Figure 1:**
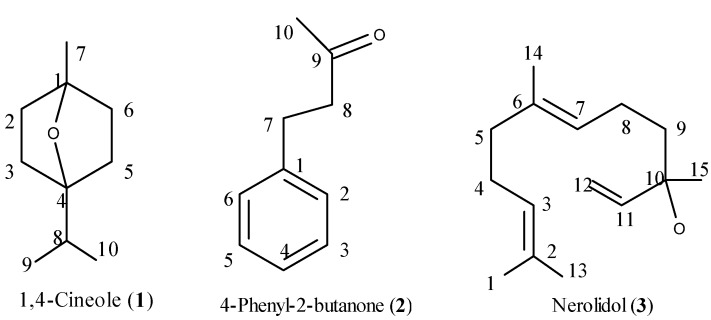
Structures of active compounds isolated from *R. anthopogonoides* flowering aerial parts.

Three bioactive compounds were isolated and based on bioassay-guided fractionation and identified based on their spectroscopic data and comparison with literature values. Their chemical structures are given in Figure 1.

Among the five major components of *R. anthopogonoides* essential oil, only three of them have been isolated. Caryophyllene and γ-elemene were not isolated in the present study by using bioactivity-guided fractionation. It maybe the concentrations used to test fractionation was too low.

### 2.2. Chemical Constituents of the Essential Oil

The oil yield of *R. anthopogonoides* flowering aerial parts was 0.86% v/w and the density of the concentrated extract was determined to be 0.92 g/mL. The GC-MS results for *R. anthopogonoides* essential oil are presented in Table 1. A total of 37 components were identified in the essential oil, accounting for 95.03% of the total oil (Table 1) and the main constituents of the essential oil were 4-phenyl-2-butanone (27.22%), nerolidol (8.08%), 1,4-cineole (7.85%), caryophyllene (7.63%) and γ-elemene (6.10%), followed by α-farnesene (4.40%) and spathulenol (4.19%). The chemical composition of the essential oil was different from that reported in other studies. For example, 4-phenyl-2-butanone (50%) was the main constituent of *R. anthopogonoides* (leaves and young twigs, collected from Datong, Qinghai Province, 36.92° N latitude and 101.67° E longitude) essential oil, followed by selina-3,7(11)-diene (20%) and neofuranodiene (10%) [24], while Li *et al.* [25] found that the essential oil of *R. anthopogonoides* (also harvested from Datong, Qinghai Province) contained4-phenyl-2-butanone (52.16%), D-limonene (4.81%), eudesma-3,7(13)-diene (4.34%), and β-myrcene (4.19%). However, methyl palmitate (17.08%), dibutyl phthalate (15.77%) and γ-cadinene (5.33%) were the main constituents of *R. anthopogonoides* young aerial parts, harvested from Guide, Qinghai Province (36.04° N latitude and 101.43° E longitude) essential oil [23]. The essential oil of *R. anthopogonoides* (aerial parts, collected from Tianzhu, Gansu Province, 37.24° N latitude and 102.84° E longitude) contained 3,7-cyclodecane-1-one (15.53%), 4-phenyl-2-butanone (13.11%), 1-propoxy-1,3-dimethoxylbene (10.80%), *O*-(*O*-methoxyphenoxy) phenol (6.65%) and 4α-methyldecahydro-naphthalene (5.94%). The above results show the wide variation in chemical composition and yield of *R. anthopogonoides* essential oil, which may be related to the herbal source (climate, soil conditions and geographical location), herbal parts used, collection time (seasonal factors), chemotype of the plant species, and/or the analytical methods used. However, 4-phenyl-2-butanone was demonstrated to be one of major constituents of the essential oil in all the previous reports, as well as in the present experiments. Moreover, the above findings suggest that further studies on plant cultivation and essential oil standardization are needed because the chemical composition of the essential oil of *R. anthopogonoides* varies greatly with the plant population.

**Table 1:** Chemical composition of the essential oil of *R. anthopogonoides* flowering aerial parts.

Compounds	RI *	Relative content (%)
β-Thujene	920	2.38
α-Pinene	939	0.78
Camphene	954	0.69
β-Pinene	974	0.22
β-Myrcene	991	0.54
1,4-Cineole	1018	7.85
(+)-Limonene	1029	1.17
1,8-Cineole	1031	0.98
(*E*)-β-Ocimene	1068	0.24
Linalool	1097	0.31
4-Terpineol	1177	1.48
α-Terpineol	1188	0.97
4-Phenyl-2-butanone	1218	27.22
γ-Pyronene	1338	0.12
Longipinene	1350	0.14
*α*-Ylangene	1370	1.41
α-Copaene	1375	1.59
β-Elemene	1389	0.27
(*Z*)-Caryophyllene	1409	1.79
α-Gurjunene	1411	0.81
α-Santalene	1420	0.57
Caryophyllene	1423	7.63
α-Bergamotene	1433	0.97
γ-Elemene	1437	6.10
2,3-Dimethylnaphthalene	1443	0.29
β-Farnesene	1453	0.23
1,4,7,-Cycloundecatriene, 1,5,9,9-tetramethyl-, *Z,Z,Z-*	1456	1.82
Curcumene	1481	0.63
α-Farnesene	1505	4.40
β-Sesquiphellandrene	1523	3.09
Nerolidol	1567	8.08
Dendrolasin	1571	1.13
Spathulenol	1578	4.19
(+)-Viridiflorol	1588	0.55
*trans*-β-Elemenone	1597	1.83
α-Santalol	1681	2.32
Total		95.03

* RI, retention index as determined on a HP-5MS column using the homologous series of *n*-hydrocarbons as reference.

### 2.3. Contact and Fumigant Toxicity

4-Phenyl-2-butanone shows pronounced contact toxicity against *S. zeamais* (LD_50_ = 6.98 μg/adult) while the crude essential oil had a LD_50_ value of 11.67 μg/adult (Table 2). 1,4-Cineole and nerolidol also exhibited contact toxicity against *S. zeamais* (LD_50_ = 50.86 μg/adult, and 29.30 μg/adult, respectively). Only 4-phenyl-2-butanone exhibits stronger contact toxicity than the crude essential oil against the maize weevils. 1,4-Cineole and nerolidol exhibit 4- and 2-times less activity against *S. zeamais*, respectively*.* The above results indicated that constituent compounds may have synergistic action or compound(s) with stronger toxicity in the essential oil were not isolated. However, when compared with the famous botanical insecticide, pyrethrum extract (25% pyrethrine I and pyrethrine II), 4-phenyl-2-butanone exhibits the same level of contact toxicity against the maize weevils and the essential oils were almost 2 times less active against *S. zeamais* adults because pyrethrum extract displayed a LD_50_ value of 4.87 μg/adult (Table 2). Compared with pyrethrum extract, 1,4-cineole and nerolidol were 10- and 6-times less active against *S. zeamais*, respectively.

**Table 2:** Toxicity of compounds isolated from *R. anthopogonoides* flowering aerial partsagainst *S. zeamais* adults.

Compounds	Contact Toxicity			Fumigant Toxicity		
	7 d LD_50_ (μg/adult) (95% FL)	Slope ± SE	Chi square (χ^2^ )	7 d LC_50_ (mg/L) (95% FL)	Slope ± SE	Chi Square (χ^2^ )
4-Phenyl-2-butanone	6.98 (6.63–7.36)	8.22 ± 0.92	13.12	3.80 (3.48–4.20)	6.67 ± 0.49	14.95
1,4-Cineole	50.86 (46.14–56.62)	4.01 ± 0.41	19.32	21.43 (20.64–25.04)	4.14 ± 0.47	16.52
Nerolidol	29.30 (26.54–31.86)	4.52 ± 0.49	22.68	>353.00	-	-
Crude oil	11.67 (10.98–12.84)	5.99 ± 0.61	11.96	9.66 (8.79–10.64)	4.43 ± 0.50	13.80
Pyrethrum extract	4.87 (4.36–5.32)	0.73 ± 0.021	13.51	-	-	-
MeBr *	-			0.67	-	-

* data from Liu and Ho [28].

4-Phenyl-2-butanone and 1,4-cineole also possess fumigant toxicity against *S. zeamais* (LC_50_ = 3.80 mg/L, and 21.43 mg/L, respectively), while the crude essential oil showed a LC_50_ value of 9.66 mg/L (Table 2) and nerolidol did not exhibit any fumigant toxicity against *S. zeamais* at the concentrations tested. 4-Phenyl-2-butanone was a bioactive (fumigant) constituent in the essential oil because it was 2.5 times more toxic to the maize weevils than the crude essential oil. The commercial grain fumigant, methyl bromide (MeBr) was reported to have fumigant activity against *S. zeamais* adults with a LC_50_ value of 0.67 mg/L [28], thus the isolated constituent 4-phenyl-2-butanone and the essential oil were 6- or 14-times less toxic to *S. zeamais* adults compared with MeBr. However, compared with other essential oils reported in the literature, the essential oil of *R. anthopogonoides* flowering aerial parts exhibited stronger than or the same level of fumigant toxicity against the maize weevils, e.g., essential oils of *Murraya exotica* (LC_50_ = 8.29 mg/L) [31], *Artemisia lavandulaefolia* (LC_50_ = 11.2 mg/L) [30], *A. vestita* (LC_50_ = 13.42 mg/L) [32], *Illicium simonsii* (LC_50_ = 14.95 mg/L) [32], *A. sieversiana* (LC_50_ = 15.0 mg/L) [30], and *Ostericum sieboldii* (20.92 mg/L) [34].

Considering the currently used fumigants are synthetic insecticides, the fumigant activity of the crude essential oil and the two isolated compounds are quite promising and they show potential for development as possible natural fumigants for the control of stored product insects. For the practical use of *R. anthopogonoides* essential oil and their constituents as novel fumigants or insecticides to proceed, further research is needed to establish their human safety and to evaluate the toxicity against other species. Additionally, their fumigant and insecticide modes of action need to be established and formulations for improving insecticidal potency and stability, thereby reducing costs, need to be developed.

## 3. Experimental

### 3.1. General

^1^H-NMR spectra were recorded on Bruker (Fallanden, Switzerland) ACF300 [300 MHz (**^1^**H)] and AMX500 [300 MHz (**^1^**H)] instruments using deuterochloroform (CDCl_3_) as the solvent with tetramethylsilane (TMS) as the internal standard. Electron impact mass spectra (EIMS) were determined on a ThermoQuest Trace 2000 mass spectrometer at 70 eV (probe). Silica gel (160–200 mesh) and pre-coated GF_254_ plates were purchased from Qingdao Marine Chemical Plant (Shandong Province, China). Fluon was purchased from ICI America Inc (Bridgewater, NJ, USA). C_8_–C_24_ *n*-alkanes were purchased from Sigma-Aldrich (USA). All other chemicals and reagents were of analytical grade.

### 3.2. Plant Material

Fresh flowering aerial parts (10 kg of leaves, stems and flowers) of *R. anthopogonoides* were harvested in July 2009 from Guide (36.04° N latitude and 101.43° E longitude), Qinghai Province, China. The aerial parts were air-dried for one week and ground to a powder. The plant species was identified and the voucher specimens (CAU-liuzhilong-2009-07-09-011) were deposited at the Department of Entomology, China Agricultural University. The ground powder of *R. anthopogonoides* was subjected to hydrodistillation using a modified Clevenger-type apparatus for 6 h and extracted with *n*-hexane. Anhydrous sodium sulphate was used to remove water after extraction. Essential oil was stored in an airtight container in a refrigerator at 4 °C.

### 3.3. Insects

The maize weevils (*S. zeamais*) were obtained from laboratory cultures maintained in the dark in incubators at 28–30 °C and 70%–80% relative humidity. The insects were reared on whole wheat at 12%–13% moisture content. Unsexed adult weevils used in all the experiments were about 2 weeks old.

### 3.4. Gas Chromatography and Mass Spectrometry

Gas chromatographic analysis was performed on an Agilent 6890N instrument (Agilent Technologies, Santa Clara, CA, USA) equipped with a flame ionization detector and an HP-5MS (30 m × 0.25 mm × 0.25 μm) capillary column, while the essential oil components were identified on an Agilent Technologies 5973N mass spectrometer. The GC settings were as follows: The initial oven temperature was held at 60 °C for 1 min and ramped at 10 °C min^−1^ to 180 °C for 1 min, and then ramped at 20 °C min^−1^ to 280 °C for 15 min. The injector temperature was maintained at 270 °C. The samples (1 μL) were injected neat, with a split ratio of 1:10. The carrier gas was helium at flow rate of 1.0 mL min^−1^. Spectra were scanned from 20 to 550 *m/z* at 2 scans s^−1^. Most constituents were identified by gas chromatography by comparison of their retention indices with those of the literature [22,23,24,25,26] or with those of authentic compounds available in our laboratories. The retention indices were determined in relation to a homologous series of *n*-alkanes (C_8_–C_24_) under the same operating conditions. Further identification was made by comparison of their mass spectra with those stored in NIST 05 and Wiley 275 libraries or with mass spectra from the literature [35]. Component relative percentages were calculated based on GC peak areas without using correction factors.

### 3.5. Contact Toxicity Using Topical Application

The contact toxicity of the essential oil and isolated compounds against *S. zeamais* adults was measured as described by Liu and Ho [28]. Range-finding studies were run to determine the appropriate testing concentrations. A serial dilution of the essential oil/compounds (15.0%–1.0% for *S. zeamias*, v/w, five concentrations) was prepared in *n*-hexane. Initial experiments were conducted to determine appropriate ranges of testing concentrations. Aliquots (0.5 μL) of the dilutions were applied topically to the dorsal thorax of the insects. Controls were determined using *n*-hexane. Both treated and control insects were then transferred to glass vials (10 insects/vial) with culture media and kept in incubators at 29–30 °C, 70%–80% r.h. Mortality of insects was observed daily until end-point mortality was reached one week after treatment. The experiments were repeated in three times. The LD_50_ values were calculated by using Probit analysis [29]. The positive control, pyrethrum extract (25% pyrethrine I and pyrethrine II), was purchased from Fluka Chemie.

### 3.6. Fumigant Toxicity

The fumigant activity of the essential oil and the pure compounds against *S. zeamais* adults was tested as described by Liu and Ho [28]. A serial dilution of the essential oil and isolated compounds was prepared in *n*-hexane. Filter papers (Whatman Cat No. 1001020, diameter 2.0 cm) were each impregnated with 20 L of an appropriate concentration (25.0%–1.0% for *S. zeamias*, v/w, five concentrations) of the essential oil/compounds, and placed on the underside of the screw cap of a glass vial (diameter 2.5 cm, height 5.5 cm, 25 mL). Preliminary range finding studies were performed with each of the compounds/essential oil to determine the appropriate testing concentrations. Fluon was used inside glass vial to prevent insects from the treated filter paper. The solvent was allowed to evaporate for 30 s before the cap was screwed tightly on the glass vial containing 10 insects.Preliminary experiments demonstrated that 30 s was sufficient for the evaporation of solvents. *n*-Hexane was used as a control. Five replicates were carried out for all treatments and controls, and they were incubated for 24 h. The insects were then transferred to clean vials with some culture media and returned to the incubator and observed daily for determination of end-point mortality, which was reached after one week. The experiments were repeated in three times. The LC_50_ values were calculated using Probit analysis [29].

### 3.7. Isolation of Active Ingredients

The crude essential oil (25 mL) was chromatographed on a silica gel (Merck 9,385, 1,000g, Merck Chemicals Co., Ltd., Shanghai, China) column (85 mm i.d., 850 mm length) by gradient elution with a mixture of solvents (*n*-hexane, *n*-hexane-ethyl acetate, and acetone). Fractions of 500 mL were collected and concentrated at 40 °C, and similar fractions according to TLC profiles were combined to yield 28 fractions. Fractions (7, 11 and 19) that possessed contact/fumigant toxicity, with similar TLC profiles, were pooled and further purified by preparative silica gel column chromatography (PTLC) until to obtain three pure compounds for determining structure as 1,4-cineole (**1**, 1.2 g), 4-phenyl-2-butanone (**2**, 1.7 g) and nerolidol (**3**, 1.9 g). The structures of the compounds were elucidated based on high-resolution electron impact mass spectrometry and nuclear magnetic resonance.

### 3.8. Compound Characterization

1,4-Cineole (**1**), colorless oil. EI-MS *m/z* (%): 154 (26), 125 (29), 111 (73), 71 (60), 69 (35), 55 (41), 43 (100), 41 (44), 27 (22). C_10_H_18_O. ^1^H-NMR (500 MHz, CDCl_3_) δ (ppm): 0.93 (3H, s, 9-CH_3_), 0.94 (3H, s, 10-CH_3_), 1.42 (3H, s, 7-CH_3_), 1.48-1.67 (8H, m, 2-CH_2_, 3-CH_2_, 5-CH_2_, 6-CH_2_), 2.04 (1H, m, *J* = 13.7, 6.8 Hz, H-8). ^13^C-NMR (125 MHz, CDCl_3_) δ (ppm): 18.12 (C-9), 18.18 (C-10), 21.22 (C-7), 32.92 (C-3, C-5), 33.06 (C-2, C-6), 37.22 (C-8), 82.96 (C-1), 89.68 (C-4). The data matched with the previous reports [36,37].

4-Phenyl-2-butanone (**2**), colorless oil, EI-MS *m/z* (%): 149 (8), 148 (73), 133 (14), 105 (81), 104 (11), 91 (60), 79 (13), 78 (11), 77 (17), 43 (100), 51 (11). C_10_H_12_O. ^1^H-NMR (500 MHz, CDCl_3_) δ (ppm): 2.11 (3H, s, 9-CH_3_), 2.73 (2H, t, *J* = 8.0 Hz, 7-CH_2_), 2.90 (2H, t, *J* = 8.0 Hz, 8-CH_2_), 7.19 (1H, dd, *J* = 8.0 Hz, 4-H), 7.21 (2H, d, *J* = 12.0, 8.0 Hz, H-2, H-6), 7.30 (2H, dd, *J* = 12.0, 8.0 Hz, H-3, H-5). ^13^C-NMR (125 MHz, CDCl_3_) δ (ppm): 29.74 (C-10), 29.95 (C-7), 45.02 (C-8), 126.12 (C-4), 128.35 (C-3, C-5), 128.51 (C-2, C-6), 141.10 (C-1), 207.62 (C-9). The ^1^H and ^13^C-NMR data were in agreement with the reported data [38,39].

Nerolidol (**3**), colorless oil. EI-MS *m/z* (%): 204 (3), 161 (11), 136 (15), 107 (24), 93 (50), 81 (27), 71 (37), 69 (100), 55 (26), 43 (28), 41 (65). C_15_H_26_O. ^1^H-NMR (500 MHz, CDCl_3_) δ (ppm): 1.29 (3H, s, 15-CH_3_), 1.56 (2H, m, *J* = 9.8, 6.1 Hz, 9-CH_2_), 1.59 (3H, s, 1-CH_3_), 1.60 (3H, s, 13-CH_3_), 1.69 (3H, s, 14-CH_3_), 1.76 (1H, br, 10-OH), 1.97–2.09 (6H, 4-CH_2_, 5-CH_2_, 8-CH_2_), 5.04–5.12 (2H, m, 12-CH_2_), 5.15 (1H, t, 3-H), 5.22 (1H, dd, *J* = 17.3, 1.2 Hz, 7-H), 5.92 (1H, m, *J* = 17.3, 10.7 Hz, 11-H). ^13^C-NMR (125 MHz, CDCl_3_) δ (ppm): 16.01 (C-14), 17.69 (C-8), 22.72 (C-1), 25.71 (C-4), 26.63 (C-13), 27.86 (C-15), 39.70 (C-5), 42.04 (C-9), 73.49 (C-10), 111.67 (C-12), 124.22 (C-3), 124.24 (C-7), 131.41 (C-2), 135.52 (C-6), 145.05 (C-11). The data matched with the previous reports [40].

## 4. Conclusions

Based on mass screening of medicinal herbs, the essential oil of *R. anthopogonoides* flowering aerial parts was found to possess toxicity against the maize weevils (*S. zeamais*). Three active constituents were isolated from the oil by bioactivity-guided fractionation and identified. 4-Phenyl-2-butanone exhibits the same level of contact toxicity against the maize weevils and the essential oils were 2-times less active against *S. zeamais* adults when compared with pyrethrum extract. Moreover, the isolated constituent 4-phenyl-2-butanone and the essential oil exhibit strong fumigant toxicity against *S. zeamais* adults, although they were 6- or 14-times less toxic when compared with MeBr. These findings suggest that essential oil of *R. anthopogonoides* and the three compounds may have potential to be developed as new natural fumigants/insecticides for the control stored product insects.
